# Synthesis of novel and stable g-C_3_N_4_-Bi_2_WO_6_ hybrid nanocomposites and their enhanced photocatalytic activity under visible light irradiation

**DOI:** 10.1098/rsos.171419

**Published:** 2018-03-28

**Authors:** Haitao Li, Na Li, Ming Wang, Beiping Zhao, Fei Long

**Affiliations:** 1 College of Material Science and Engineering, Guilin University of Technology, Guilin 541004, People's Republic of China; 2 Guangxi Ministry-Province Jointly-Constructed Cultivation Base for State Key Laboratory of Processing for Nonferrous Metal and Featured Materials, Guilin University of Technology, Guilin 541004, People's Republic of China

**Keywords:** g-C_3_N_4_, Bi_2_WO_6_, composite materials, visible light responded photocatalysts

## Abstract

Graphitic carbon nitride (g-C_3_N_4_) nanosheets with a thickness of only a few nanometres were obtained by a facile deammoniation treatment of bulk g-C_3_N_4_ and were further hybridized with Bi_2_WO_6_ nanoparticles on the surface via a solvothermal method. The composite photocatalysts were characterized by powder X-ray diffraction, scanning electron microscopy, transmission electron microscopy, UV–vis diffuse reflection spectroscopy and X-ray photoelectron spectroscopy (XPS). The HR-TEM results show that the nano-sized Bi_2_WO_6_ particles were finely distributed on g-C_3_N_4_ sheet surface, which forms heterojunction structure. The UV–vis diffuse reflectance spectra (DRS) show that the absorption edge of composite photocatalysts shifts towards lower energy region in comparison with those of pure g-C_3_N_4_ and Bi_2_WO_6_. The degradation of methyl orange (MO) tests reveals that the optimum activity of 8 : 2 g-C_3_N_4_-Bi_2_WO_6_ photocatalyst is almost 2.7 and 8.5 times higher than those of individual g-C_3_N_4_ and Bi_2_WO_6_. Moreover, the recycle experiments depict high stability of the composite photocatalysts. Through the study of the influencing factors, a possible photocatalytic mechanism is proposed. The enhancement in both photocatalytic performance and stability was caused by the synergistic effect, including the effective separation of the photogenerated electron-hole pairs at the interface of g-C_3_N_4_ and Bi_2_WO_6_, the smaller the particle size and the relatively larger specific surface area of the composite photocatalyst.

## 1. Introduction

Semiconductor photocatalysts have drawn much attention in the past decades because they represent a promising technology to use natural sunlight energy to promote chemical reactions, such as water-splitting, pollutant degradation and organic transformation [1,2]. Up to now, a mass of oxide and sulfide semiconductor photocatalysts have been developed for photocatalytic reactions, for example, the extensively investigated titanium dioxide and zinc sulfide [2,3]. However, the traditional photocatalysts are active only in the UV region and have high electron-hole recombination rates, which led to its inability to make full use of solar energy and reduce the photocatalytic performance [4]. Thus, it is still a challenging task looking for the sustainable, high-efficiency and visible-light-responsive photocatalytic materials.

Recently, non-metallic polymer photocatalyst g-C_3_N_4_, which shows superior photocatalytic activity for hydrogen production through water-splitting under visible light irradiation, was reported by Wang *et al.* [5]. Unlike traditional wide band-gap photocatalysts, this novel g-C_3_N_4_ photocatalyst possesses a narrow band gap (2.71 eV) and favourable thermal and chemical stability, which make it a unique electronic structure and highly condensed [5]. In addition, carbon and nitrogen elements, the composition of g-C_3_N_4_, are abundant in natural source. All these merits of g-C_3_N_4_ make it a valuable photocatalyst for solar energy-driven applications. However, the photocatalytic activity of g-C_3_N_4_ is still restricted due to the high electron-hole recombination rate and low specific surface area (generally below 10 m^2^ g^–1^ for the bulk g-C_3_N_4_) [6]. To further improve the photocatalytic activity of g-C_3_N_4_, many strategies have been proposed, including element doping, designing a textural porosity, as well as coupling g-C_3_N_4_ with heterogeneous semiconductor composites [7–9].

To reduce the recombination rates of photogenerated electron-hole pairs, construction of heterojunctions by coupling of two semiconductors was proved to be an effective strategy. Up to now, several g-C_3_N_4_-based heterojunction composites have been reported, for instance, g-C_3_N_4_-TiO_2_, g-C_3_N_4_-ZnO, g-C_3_N_4_-CdS, g-C_3_N_4_-BiOBr and g-C_3_N_4_-TaON [10–14]. All these composites exhibit enhanced photocatalytic performance with respect to the sole component, which was ascribed to the effective separation of charged carriers. Considering the band structures of two coupled semiconductors, three types of heterojunction structures are usually formed. Type II heterojunction was regarded as the most effective band structure to separate charged carriers, in which the electrons transfer from one semiconductor to the other, while the holes migrate reversely [15]. According to the conduction band (CB) and valence band (VB) potential value of g-C_3_N_4_ (−1.13 and 1.58 eV, respectively), it was considered that the Bi_2_WO_6_ (0.46 and 3.26 eV, respectively) is one of the most suitable components to form type II heterojunction with g-C_3_N_4_ based on energy levels [16–18]. Furthermore, the crystal structure of Bi_2_WO_6_ comprises accumulated layers of alternating bismuth oxide (Bi_2_O_2_)^2+^ and tungsten oxide (WO_4_)^2−^ sheets, which is favourable for charge transfer in plane.

This paper presents a facile strategy to produce hybrid g-C_3_N_4_ nanosheets with Bi_2_WO_6_ nanoparticles by two steps. First, bulk g-C_3_N_4_ was synthesized by thermal polycondensation of melamine. To make the specific surface area of g-C_3_N_4_ larger, the bulk g-C_3_N_4_ was treated by a simple deammoniation process at 500°C for 3 h, and then the g-C_3_N_4_ nanosheets with several nanometres thickness were obtained. Second, using g-C_3_N_4_ nanosheets as bases, Bi_2_WO_6_ nanoparticles were deposited onto g-C_3_N_4_ surface by a solvothermal process and a series of composite photocatalysts with different weight ratio were prepared. It was found that the two components coexisted and closely constructed a heterojunction structure. Furthermore, owing to the larger specific surface area and well-matched band structures, the activity of the obtained composed catalysts was significantly high than those of the pure Bi_2_WO_6_ and g-C_3_N_4_ respectively. In addition, these composed catalysts were very stable and could be used multiple times, retaining a relatively high photocatalytic activity. Finally, through the analysis of the capture experiment, the possible photocatalytic mechanism was put forward.

## 2. Experimental set-up

### 2.1. Synthesis

All chemicals used were commercially available and used without further purification. Bulk g-C_3_N_4_ was prepared by the thermal treatment of 4 g melamine at 550°C for 4 h in a muffle furnace under an ambient pressure. Further deammoniation treatment was performed at 500°C for 3 h [19,20]. After reaction, the alumina crucible was cooled to room temperature. The resultant yellow product was collected for further use.

The g-C_3_N_4_-Bi_2_WO_6_ composite photocatalysts were obtained by hybridization of g-C_3_N_4_ nanosheet with Bi_2_WO_6_ nanoparticles through a solvothermal method. In a typical procedure, a certain amount of g-C_3_N_4_ sheets were added into 30 ml triethylene glycol (TEG) and sonicated for 30 min, and then a certain percentage of Bi(NO_3_)_3_·5H_2_O dissolved in 20 ml TEG by stirring and added dropwise to the above suspension. The mixture was stirred for 1.5 h at room temperature to form a homogeneous solution. Meanwhile, a certain amount of Na_2_WO_4_·2H_2_O solid was added to 20 ml ethylene glycol (EG) to obtain a uniform solution. After stirring and dissolving, the solution was added rapidly to the above mixture suspension and then stirred for another 3 h at room temperature. After that, the solution was transferred into a Teflon-lined steel autoclave and kept at 200°C for 12 h. Subsequently, the precipitate was collected by centrifugal separation, washed with distilled water and ethanol several times, and then dried at 60°C for 12 h. Finally, the obtained g-C_3_N_4_-Bi_2_WO_6_ photocatalysts were ground for further use. In the same procedure, different mass ratios of g-C_3_N_4_-Bi_2_WO_6_ at 2 : 8, 5 : 5 and 8 : 2 were prepared and denoted as 2 : 8 g-C_3_N_4_-Bi_2_WO_6_, 5 : 5 g-C_3_N_4_-Bi_2_WO_6_ and 8 : 2 g-C_3_N_4_-Bi_2_WO_6_, respectively. The pure Bi_2_WO_6_ sample was synthesized according to the same method without g-C_3_N_4_.

### 2.2. Characterization

XRD patterns were recorded on a PANalytical X'pert PRO powder diffractometer equipped with a Cu K*α* radiation source (*λ* = 0.15405 nm). FT-IR spectra were measured on a Thermo Electron Nicolet-Nexus 470 FT-IR spectrometer (KBr disc). X-ray photoelectron spectroscopy (XPS) of the photocatalysts was performed on an ESCALAB 250Xi (Thermo Electron Corporation, USA). SEM was employed to observe the morphology of samples (Hitachi S-4800 microscope). TEM and HR-TEM were applied to characterize the microstructure of the samples (JEOL, JEM-2100). UV−vis diffuse reflectance spectra (DRS) were measured using a UV−vis spectrometer (Shimadzu, UV-3600, Japan). The photoluminescence (PL) spectra of the photocatalysts were obtained by a VARIAN fluorescence spectrophotometer.

### 2.3. Photocatalytic tests

The photocatalytic activity of the catalysts was tested by degrading the methyl orange (MO) solution under a visible light using a 500 W xenon lamp with a 420 nm cut-off filter as the light source. The specific test procedure was as follows: 50 mg of the sample was dispersed in 50 ml of methyl orange solution (5 mg l^−1^) and stirred for 120 min under dark conditions to achieve adsorption equilibrium. After the start of the experiment, for a certain time period, about 3 ml suspensions were collected and centrifuged to wipe off the photocatalytics. The concentrations of residued MO were then monitored by a UV–vis spectrometer at a wavelength of 463 nm.

## 3. Results and discussions

### 3.1. Characterization

**Figure 1.**
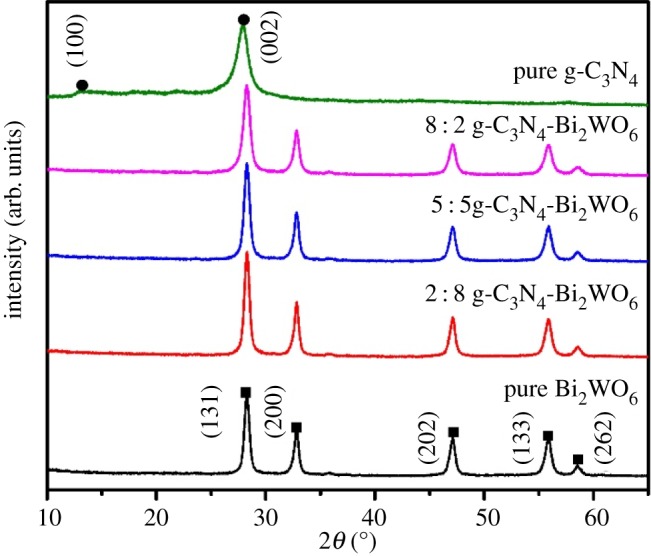
XRD patterns of as-synthesized g-C_3_N_4_, Bi_2_WO_6_ and g-C_3_N_4_-Bi_2_WO_6_ photocatalysts.

Figure 1 shows the XRD patterns of the as-prepared samples. The pure g-C_3_N_4_ sample shows two diffraction peaks at 12.97°and 27.93°, corresponding to the (100) and (002) planes, which are attributed to the periodic arrangement of the basic structural units in the g-C_3_N_4_ and the interlayer build-up of the cyclic aromatic substance [6]. For pure Bi_2_WO_6_, there is a series of narrow and pointed diffraction peaks, which are in good agreement with the orthorhombic phase of Bi_2_WO_6_ (JCPDS 39-0256) without any impurity. After the two materials are compounded, the g-C_3_N_4_-Bi_2_WO_6_ composite was similar to the pure Bi_2_WO_6_ diffraction pattern. This phenomenon can be ascribed to two aspects: one is that the (002) of g-C_3_N_4_ and (131) of Bi_2_WO_6_ diffraction peaks were located in a similar position (approx. 28°), and the other is that the g-C_3_N_4_ layer was too thin, thus the diffraction intensity is relatively weak with respect to those of Bi_2_WO_6,_ which make g-C_3_N_4_ diffractions invisible in the composite [21].

**Figure 2.**
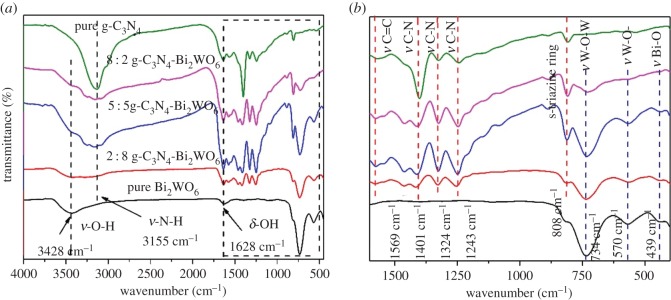
(*a*–*b*) FT-IR spectra of the as-synthesized g-C_3_N_4_, Bi_2_WO_6_ and g-C_3_N_4_-Bi_2_WO_6_.

The FT-IR spectra of g-C_3_N_4_, Bi_2_WO_6_ and g-C_3_N_4_-Bi_2_WO_6_ photocatalysts are shown in figure 2*a*. The absorption peaks at 3428 cm^−1^ and 1628 cm^−1^ in the spectrum of pure Bi_2_WO_6_ are attributed to the stretching vibration and bending vibration of the O–H, respectively. For g-C_3_N_4_ sample, the 3155 cm^−1^ band can be attributed to the N–H stretching mode. The enlarged spectrum from 400 to 1600 cm^−1^ is shown in figure 2*b*. The main absorption peaks at 400–800 cm^−1^ in pure Bi_2_WO_6_ sample correspond to Bi–O, W–O stretching and W–O–W bridging stretching modes [22]. In the case of pure g-C_3_N_4_, the intense band at 808 cm^−1^ belongs to the characteristic breathing mode of s-triazine, and the strong bands in the 1200–1600 cm^−1^ region with peaks at 1243, 1324, 1401 and 1569 cm^−1^ correspond to typical stretching vibration modes of C=N and C–N heterocycles [23,24]. All these peaks can be found in g-C_3_N_4_-Bi_2_WO_6_ photocatalyst, indicating the presence of g-C_3_N_4_ and Bi_2_WO_6_ components in composite photocatalysts.

**Figure 3.**
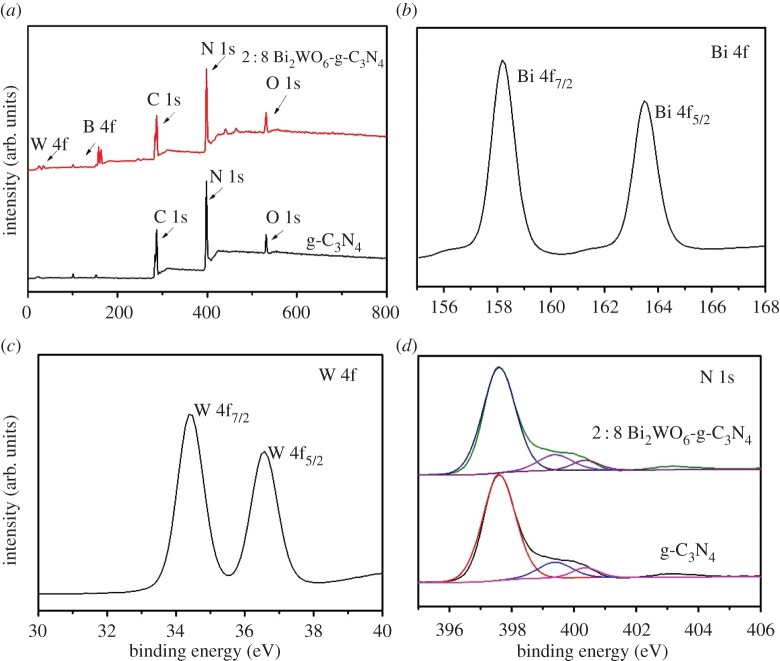
XPS spectra of the photocatalysts: (*a*) whole XPS spectra of g-C_3_N_4_ and 8 : 2 g-C_3_N_4_-Bi_2_WO_6_ composite, (*b*) Bi 4f and (*c*) W 4f of 8 : 2 g-C_3_N_4_-Bi_2_WO_6_ composite, and (*d*) N 1s of g-C_3_N_4_ and 8 : 2 g-C_3_N_4_-Bi_2_WO_6_ composite.

The elemental composition of the samples was measured by XPS. We can see that the g-C_3_N_4_ mainly consisted of C, N and a little amount of O elements (figure 3*a*). The O element that appeared here may be ascribed to O_2_ adsorbed on the surface during polymerization process, which usually occurs in synthetic g-C_3_N_4_ materials [25]. As shown in figure 3*b*, two peaks at 157.58 and 162.88 eV can be attributed to Bi 4f_7/2_ and 4f_5/2_ of Bi^3+^ ions. The XPS of W 4f electrons is shown in figure 3*c*, indicating the + 6 valence of W element [26]. Figure 3*d* shows high-resolution N 1s spectra of the samples. The N 1s spectrum could be fitted into four peaks, corresponding to four binding energies of sp2-bonded nitrogen in C–N=C (*ca* 398.7 eV), nitrogen in tertiary N–(C)_3_ groups (*ca* 400.3 eV), amino groups C–N–H (*ca* 401.4 eV) caused by imperfect polymerization and π-excitations (*ca* 404.3 eV) [27,28]. All of these results further confirmed the coexistence of g-C_3_N_4_ and Bi_2_WO_6_ in g-C_3_N_4_-Bi_2_WO_6_ composite photocatalysts.

**Figure 4.**
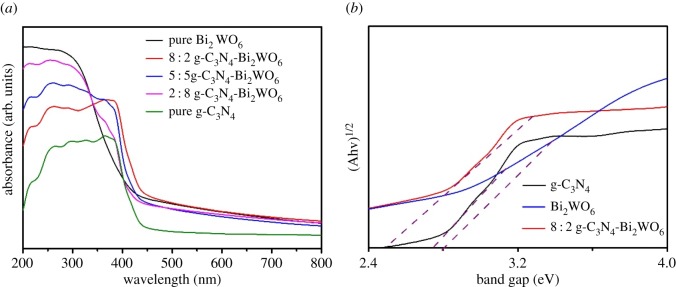
(*a*,*b*) UV–vis DRS of the as-synthesized g-C_3_N_4_, Bi_2_WO_6_ and g-C_3_N_4_-Bi_2_WO_6_ photocatalysts.

The band structures of as-prepared samples were evaluated by the UV–vis DRS technique. As shown in figure 4*a*, the pristine Bi_2_WO_6_ has an absorption edge of approximately 443 nm, which corresponds to a band gap of approximately 2.80 eV [17,29]. The absorption edge of pure g-C_3_N_4_ was at about 457 nm, corresponding to the band gap of 2.71 eV, which is consistent with the reported values in the literature [30]. After combining the two semiconductors, a gradual red shift appeared as the amount of g-C_3_N_4_ increased; this should be the result of the interaction between g-C_3_N_4_ and Bi_2_WO_6_ in the heterojunction [31,32]. The band-gap values were also estimated from the intercept of tangents to plots of (Ahv) ^1/2^ versus photon energy [31], as shown in figure 4*b*. It is worth noting that the g-C_3_N_4_-Bi_2_WO_6_ composite shows narrower band gap in comparison with pure g-C_3_N_4_ and Bi_2_WO_6_. The results indicate that the g-C_3_N_4_-Bi_2_WO_6_ heterojunction complex photocatalyst has a good visible light response, which can enhance its photocatalytic activity.

**Figure 5.**
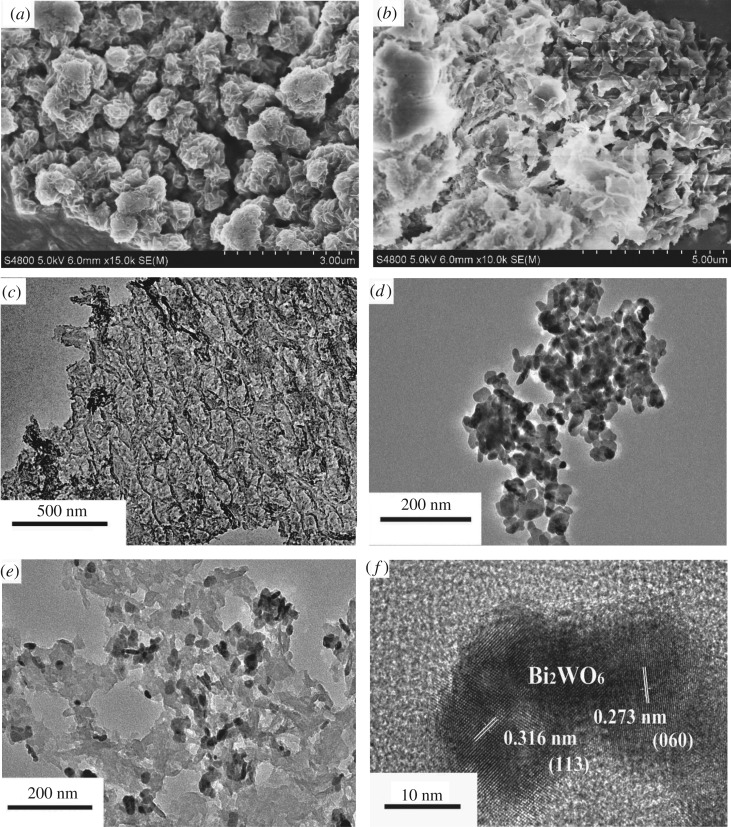
Characterization of the as-synthesized Bi_2_WO_6_ and g-C_3_N_4_ photocatalyst:(*a*) g-C_3_N_4_ and (*b*) 8 : 2 g-C_3_N_4_-Bi_2_WO_6_ composite of SEM images, (*c*) g-C_3_N_4_, (*d*) Bi_2_WO_6_ and (*e*) 8 : 2 g-C_3_N_4_-Bi_2_WO_6_ sample of TEM images, (*f*) HR-TEM image of the 8 : 2 g-C_3_N_4_-Bi_2_WO_6_ heterostructure.

As seen from the SEM image of figure 5*a*, g-C_3_N_4_ has a hierarchical flower-like morphology, which was assembled by many nanoflakes with a thickness of only a few nanometres. The nanoflake structure was further characterized by TEM observation. As shown in figure 5*c*, a typically two-dimensional lamellar structure with a thickness of several nanometres of g-C_3_N_4_ nanoflakes was observed, which is similar to the reported reference [33]. The SEM image of 8 : 2 g-C_3_N_4_-Bi_2_WO_6_ composite is exhibited in figure 5*b*. Clearly, with the coupling of Bi_2_WO_6_ nanoparticles, the hierarchical structures of g-C_3_N_4_ were disassembled into discrete nanoflakes. With the increase of Bi_2_WO_6_ content, the g-C_3_N_4_ nanoflake was completely covered by Bi_2_WO_6_ nanoparticles, which would have a negative effect on the photocatalysis because of the decrease in photocatalytic reaction site on the g-C_3_N_4_ nanoflake surface. Figure 5*d* shows the TEM image of pure Bi_2_WO_6_ sample. It can be observed that Bi_2_WO_6_ formed irregular nanoparticles with a mean size of approximately 30 nm. From the TEM image of the 8 : 2 g-C_3_N_4_-Bi_2_WO_6_ sample (figure 5*e*), we know that Bi_2_WO_6_ nanoparticles were closely covered on the g-C_3_N_4_ nanosheet surface. The HR-TEM image of figure 5*f* shows lattice fringe spacing of 0.316 and 0.273 nm, which belongs to (113) and (060) lattice planes of cubic Bi_2_WO_6_. Because of low crystallinity of the g-C_3_N_4_, it is hard to find the lattice fringe of g-C_3_N_4_ [23]. The result obviously shows that g-C_3_N_4_ nanosheets form a close heterogeneous contact with Bi_2_WO_6_. This close contact interface accelerates the migration of photogenerated electrons and holes between g-C_3_N_4_ and Bi_2_WO_6_, inhibiting the recombination of photogenerated electron-hole pairs and improving the photocatalytic activity.

### 3.2. Photocatalytic performance

**Figure 6.**
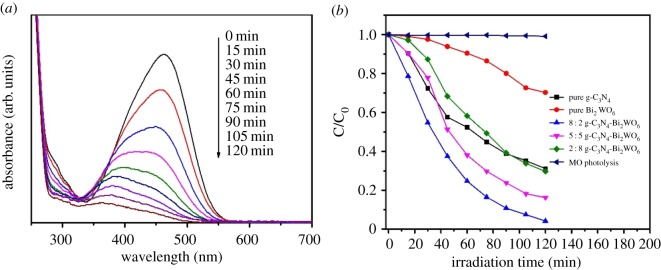
(*a*) Absorption spectra of MO with irradiation time over 8 : 2 g-C_3_N_4_-Bi_2_WO_6_ composite photocatalyst. (*b*) Degradation rates of MO under visible light irradiation without catalyst and in the presence of g-C_3_N_4_, Bi_2_WO_6_ and g-C_3_N_4_-Bi_2_WO_6_ samples.

Based on the above conclusions, we evaluated the photocatalytic activity of the synthesized samples by degrading methyl orange under visible light irradiation. As shown in figure 6*a*, the highest degradation rate of 95.88% after 120 min irradiation was obtained over 8 : 2 g-C_3_N_4_-Bi_2_WO_6_ composite sample. Meanwhile, no other peaks in the UV region indicate the full decomposition of aromatic component. To compare the photocatalytic performance of different photocatalysts, the degradation rate of MO was determined by the characteristic absorption peak of methyl orange at 463 nm. The C/C_0_ versus irradiation time is plotted in figure 6*b*. The concentration of MO solution without photocatalyst did not change after 120 min degradation, indicating that methyl orange is quite stable, and ruled out the possibility of the occurrence of its self-degradation. As the Bi_2_WO_6_ sample has a relatively high photogenerated electron-hole pairs recombination rate, the pure Bi_2_WO_6_ sample exhibits its low photocatalytic activity. Similarly, only 68.8% of MO was degraded by the g-C_3_N_4_ sample for the same irradiation time. It was expected that the construction of proper heterojunction can enhance the photocatalytic activity through effective separation of photogenerated electron-hole pairs. As proved in this paper, all the g-C_3_N_4_-Bi_2_WO_6_ composite photocatalysts present enhanced photocatalytic ability for the degradation of MO compared to pure Bi_2_WO_6_ and g-C_3_N_4_. Besides, the highest activity was obtained over 8 : 2 g-C_3_N_4_-Bi_2_WO_6_ composite, and its degradation rate was 1.4 and 3.2 times higher than that of pure g-C_3_N_4_ and Bi_2_WO_6_, respectively. It should be noted that too much Bi_2_WO_6_ content in composite photocatalyst (such as 2 : 8 g-C_3_N_4_-Bi_2_WO_6_) would distinctly reduce the photocatalytic activity of the composite. Previous research also pointed out that the dispersion and size of deposited nanoparticles could influence their photocatalytic efficiency [34,35]. On g-C_3_N_4_ nanosheets, Bi_2_WO_6_ nanoparticles with smaller size and favourable dispersibility meant higher photocatalytic activity. The smaller size and higher dispersibility of Bi_2_WO_6_ on the g-C_3_N_4_ sheets meant higher photocatalytic activity. But if the loading density of Bi_2_WO_6_ nanoparticle was too high, the photocatalytic site on the g-C_3_N_4_ sheet surface will be covered, which would damage the heterojunction structure and reduce synergistic effect between the two components [36]. As a result, only Bi_2_WO_6_ nanoparticles coated on g-C_3_N_4_ with proper size and dispersion would enhance the photocatalytic activity of composite photocatalyst. In this work, it was found the 8 : 2 g-C_3_N_4_-Bi_2_WO_6_ composite sample displayed the highest catalytic performance due to the optimal structure.

**Figure 7.**
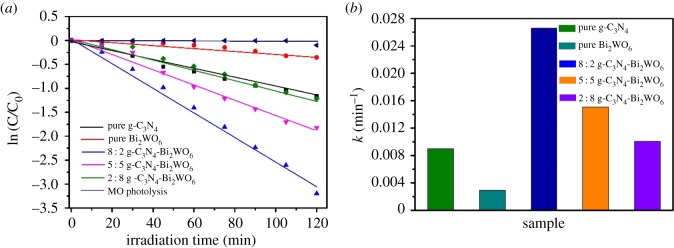
(*a*) The first-order kinetics plot and (*b*) the kinetic constants for the photodegradation of MO under visible light irradiation (*λ* > 420 nm) by g-C_3_N_4_, Bi_2_WO_6_ and g-C_3_N_4_- Bi_2_WO_6_ samples.

The kinetics of photocatalytic degradation of methyl orange reflects the reaction rate of the photocatalyst. The change of methyl orange concentration can be obtained by the first-order kinetic equation ln (C_0_ /C_t_) = *k* *t* to obtain the corresponding apparent degradation rate constant (*k*) *T*, where C_0_ and C are the concentrations of pollutant in solution at time *t*_0_ and *t*, respectively, and *k* is the apparent first-order rate constant [37]. As shown in figure 7*a*, all fitting curves of *t* for ln (C/C_0_) are approximated by a straight line, and thus the corresponding kinetic constants (*k*) are calculated. Observably, the rate constant *k* is calculated to be 0.00999, 0.01104, 0.0166, 0.02659 and 0.00313 min^−1^ for pure g-C_3_N_4_, 2 : 8 g-C_3_N_4_-Bi_2_WO_6_, 5 : 5 g-C_3_N_4_-Bi_2_WO_6_, 8 : 2 g-C_3_N_4_-Bi_2_WO_6_ and pure Bi_2_WO_6_ samples, respectively (figure 7*b*). That is, 8 : 2 g-C_3_N_4_-Bi_2_WO_6_ catalyst has the best photocatalytic activity, the degradation rate of methyl orange is almost 2.7 and 8.5 times higher than that of either individual g-C_3_N_4_ or Bi_2_WO_6_. The above results show that the introduction of Bi_2_WO_6_ could effectively enhance the visible light photocatalytic activity of g-C_3_N_4_

**Figure 8.**
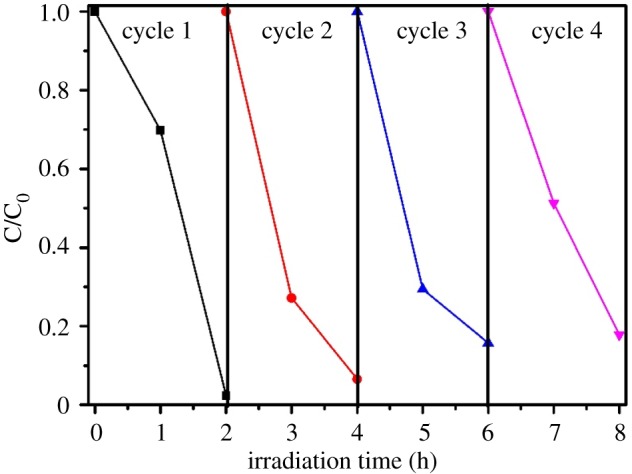
Cycling runs for the photocatalytic degradation of MO over 8 : 2 g-C_3_N_4_-Bi_2_WO_6_ composite sample under visible light irradiation.

Apart from the photocatalytic performance, the stability of photocatalysts in the practical application also has a very important significance. The g-C_3_N_4_-Bi_2_WO_6_ composite photocatalyst was circulated four times under the same conditions. After every cycle, the sample was centrifuged, washed and dried at 60°C. After four cycles, the degradation rate of methyl orange was still 84.26% (figure 8), which shows a high photocatalytic stability of the composite after four recycling runs. Thus, the stability and recyclability of g-C_3_N_4_-Bi_2_WO_6_ composites are excellent.

### 3.3. Possible photocatalytic mechanism

**Figure 9.**
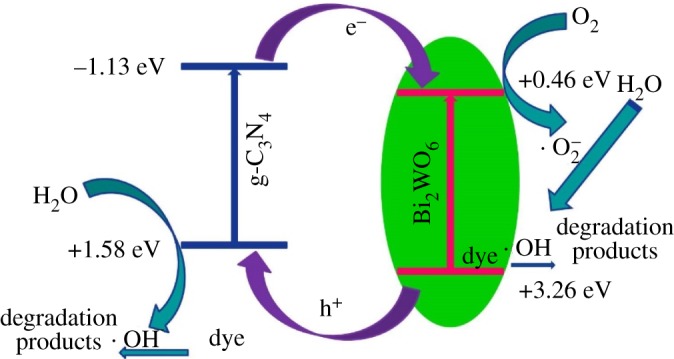
Band structure schematic of g-C_3_N_4_-Bi_2_WO_6_ heterojunction and possibly occurring reaction mechanism of MO on the surface.

It can be seen from the photocatalytic experiments that the novel g-C_3_N_4_-Bi_2_WO_6_ composite has shown a good photocatalytic effect for degrading the MO under visible light. On the basis of the above experimental results, a possible mechanism for the g-C_3_N_4_-Bi_2_WO_6_ composite photocatalyst is proposed. The process of electron-hole separation and transport in interface is shown in figure 9. It is known from the literature that the band-gap positions of g-C_3_N_4_ were determined at −1.13 and +1.58 eV [4], while those of Bi_2_WO_6_ were estimated at 0.46 and +3.26 eV. Once the composite photocatalyst is irradiated by visible light, both g-C_3_N_4_ and Bi_2_WO_6_ can be stimulated and generate photogenerated electron-hole pairs. Because it has a well-matched staggered band-gap structure and a close interface, the electrons on the conduction band of g-C_3_N_4_ will be transferred to the conduction band of Bi_2_WO_6_. At the same time, holes on the VB of Bi_2_WO_6_ reversely transfer to the VB of g-C_3_N_4_. This process effectively facilitates the separation of photogenerated electron-holes, thereby inhibiting the recombination of photogenerated electron-hole pairs, which could improve the photogenerated electron-hole pair separation effectively and decrease the possibility of photogenerated charge recombination greatly. Therefore, Bi_2_WO_6_ nanoparticles and g-C_3_N_4_ nanosheet in the g-C_3_N_4_-Bi_2_WO_6_ complex material could form the optimal heterojunction structures, which result in the improvement of the photocatalytic activity.

**Figure 10.**
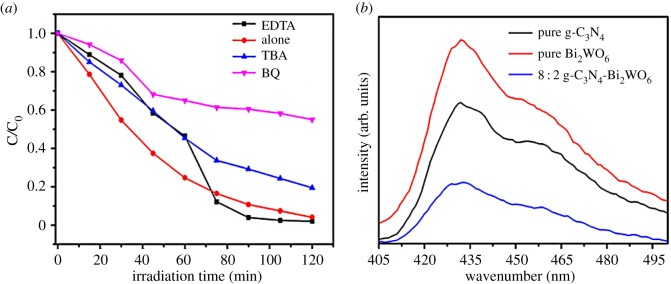
(*a*) Effects of different scavengers on degradation of MO in the presence of 8 : 2 g-C_3_N_4_-Bi_2_WO_6_ heterojunction and (*b*) PL spectra of the as-synthesized g-C_3_N_4_, Bi_2_WO_6_ and 8 : 2 g-C_3_N_4_-Bi_2_WO_6_ photocatalysts at room temperature.

As we know, photogenerated holes, ·OH radicals and ·O_2_^−^ are the three main major active species in the degradation process [13,38]. Therefore, to study the role of these reactive groups in the photocatalytic process, a series of free radicals capture experiments were conducted by using ethylenediaminetetraacetate (EDTA, 6 mmol l^−1^), benzoquinone (BQ, 0.5 mmol l^−1^) and tert-butanol (TBA, 6 mmol l^−1^) as effective scavengers for holes, ·O_2_^−^ and ·OH radicals respectively [39]. In the free radicals capture experiments (figure 10*a*), when TBA was added to the system, the degradation efficiency of MO was reduced from 95.89% to 80.53% compared with the photocatalytic experiment without the capture agent, indicating that the photogenerated holes are not the primary active group for the degradation of methyl orange, and they are also not the sources of ·OH radicals. It is worth noting that the degradation of MO is slightly enhanced when the hole trapping agent EDTA was added, which means that the hole is not the active group for degrading the MO, and the reason for the increase in catalytic activity is that EDTA is a capture agent, so that the photogenerated electron-hole could be effectively separated. However, when BO was added, the degradation rate of MO was only 45.02%, which demonstrates that the main active species should be ·O_2_^−^ in the degradation of MO [40,41].

For further insight into photogenerated electron-hole pair behaviour of the g-C_3_N_4_-Bi_2_WO_6_ composite and to verify the above-mentioned mechanism, PL spectra of the as-prepared photocatalysts were obtained. Generally, in semiconductor materials, the migration and separation of photogenerated carriers lead to the generation of fluorescence spectra [42,43]. Figure 10*b* presents the PL spectra of pure g-C_3_N_4_, pure Bi_2_WO_6_ and 8 : 2 g-C_3_N_4_-Bi_2_WO_6_ composite samples excited with a 365 nm light. At room temperature, the luminescent characteristic peaks of pure g-C_3_N_4_ are centred at 440 nm, which is attributed to the radiation recombination process of self-trapped excitations [44]. Compared with those of pure g-C_3_N_4,_ the position of the emission peaks of the 8 : 2 g-C_3_N_4_-Bi_2_WO_6_ sample was almost unchanged, but the intensity was greatly reduced, which indicates that the photogenerated charges recombination rate was controlled in the heterojunction semiconductors. The PL results support the above discussion on the photocatalytic experiments and proposed mechanism strongly. Over all, the g-C_3_N_4_-Bi_2_WO_6_ composite photocatalysts can effectively separate photogenerated electron-hole pairs and inhibit recombination of the charges, which has a very promising application in environmental purification.

## 4. Conclusion

In conclusion, the g-C_3_N_4_-Bi_2_WO_6_ composite photocatalyst with a heterojunction structure was prepared by a simple solvothermal method. This novel g-C_3_N_4_-Bi_2_WO_6_ composite catalyst exhibits outstanding visible light response photocatalysis, attributing to the effective separation of electron-hole pair at heterojunction interfaces. Among the as-prepared various weight ratios of g-C_3_N_4_-Bi_2_WO_6_ samples, the 8 : 2 g-C_3_N_4_-Bi_2_WO_6_ sample displayed best photocatalytic activity because of its optimum structure. The investigation of the photocatalytic mechanism showed that the degradation of methyl orange by the g-C_3_N_4_-Bi_2_WO_6_ sample is mainly through ·O_2_^−^ radicals, and ·OH was verified to be inappreciable. We believe this work provides some new insights for fabrication of highly efficient heterojunction photocatalysts for environmental remediation.

## Data accessibility

This paper does not contain any additional data.
